# Peer victimization and peer sexual harassment across early adolescence: Branches from the same tree or free‐standing constructs?

**DOI:** 10.1111/jora.70079

**Published:** 2025-10-03

**Authors:** Kristina Holmqvist Gattario, Andrea Valik, Carolina Lunde, Therése Skoog, Darun Jaf

**Affiliations:** ^1^ Department of Psychology University of Gothenburg Gothenburg Sweden; ^2^ School of Behavioural, Social and Legal Sciences Örebro University Örebro Sweden; ^3^ Present address: Department of Research and Development Gothenburg Sweden

**Keywords:** early adolescence, exploratory structural equation modeling, peer sexual harassment, peer victimization

## Abstract

Researchers have debated whether peer victimization and peer sexual harassment (PSH) are branches from the same tree and/or whether they are different constructs; yet no previous study has been able to clarify this issue. We used exploratory structural equation modeling to examine three different, theoretically informed ways of conceptualizing peer victimization and PSH. Annual three‐wave questionnaire data included 997 participants at T1 (*M* age = 10.0 years, *SD* = 0.3). Results indicated that peer victimization and PSH should best be viewed as two distinct, yet related constructs – a proposition valid across both time (ages 10–12) and genders. The findings from the present study can inform future research on adolescents' adverse peer experiences.

## INTRODUCTION

The literature concerning adolescents' experiences of adverse peer relations now encompasses a range of constructs in its theories and measurements. One of the most frequently examined constructs, receiving increasing attention since the 1970s, is peer victimization (Hawker & Boulton, 2000; Olweus, 1978). In the 1980s and 1990s, the construct of peer sexual harassment (PSH) began to emerge in the literature—initially examined among older adolescents and adults (Lott et al., 1982), and later among early adolescents (Roscoe et al., 1994). The emergence of this construct raised questions among researchers regarding its similarities to, and distinctions from, peer victimization (Charmaraman et al., 2013; Gruber & Fineran, 2008). While both peer victimization and PSH involve exposure to unwanted behaviors from peers (Nickerson et al., 2022) and may be considered branches of the broader construct of school‐based victimization (Marx & Kettrey, 2016), PSH—due to its gendered and sexual dimension—has also been conceptualized as a construct of its own, separate from peer victimization (Espelage et al., 2018; Gruber & Fineran, 2016). To date, however, there is no empirical research that has used the appropriate analytical tools and designs to achieve clarity regarding whether peer victimization and PSH are in fact branches from the same tree and/or whether they are different constructs. In this study, we used exploratory structural equation modeling (ESEM), an analytical method that has been developed (Asparouhov & Muthén, 2009; Marsh et al., 2014) and used across different areas of research to examine the factorial structure and psychometric properties of various constructs (Fadda et al., 2019; Swami et al., 2023). Hence, building on current theoretical and empirical knowledge, and applying the ESEM framework, we empirically examine the level of overlap and uniqueness in items measuring peer victimization and PSH. Further, we test whether the factorial structure of peer victimization and PSH is consistent across both time (ages 10–12 years) and genders. The study provides novel knowledge to inform how PSH should be viewed in relation to peer victimization and thereby inform future research and interventions on both peer victimization and PSH.

### Defining peer victimization and peer sexual harassment

Peer victimization involves peers' intentional acts of aggression that victims perceive as harmful (Card & Hodges, 2008). Being exposed to peer victimization may include being called nasty names, being teased, threatened, socially excluded, hit, pushed, or kicked (Hawker & Boulton, 2000). Factorial analyses (confirmatory factor analysis [CFA] and ESEM) suggest that peer victimization comprises three dimensions: relational, verbal, and physical victimization (Marsh, Guo, et al., 2023). It is worth noting that the construct of bullying (involving an intention to harm, repetitive actions, and an imbalance of power between the bully and the victim; Olweus, 1993) is sometimes used interchangeably with peer victimization. In fact, a sizable proportion of bullying research likely captures peer victimization instead of bullying, since bullying measurements often do not fully assess the criteria of bullying (e.g., imbalance of power; Bjereld et al., 2020; Jia & Mikami, 2018). For the sake of clarity, we will use the broader term of peer victimization as the reference construct to which we aim to compare and understand PSH. However, because peer victimization and bullying are to some extent overlapping constructs (Bjereld et al., 2020; Jia & Mikami, 2018), we will make use of research related to bullying when arguing for similarities and differences between the constructs of peer victimization and PSH.

PSH has been defined as being the target of “unwanted sexual attention” from peers (McMaster et al., 2002, p. 92). As such, PSH may include being exposed to uninvited sexual comments, homophobic and misogynistic name‐calling, unwanted grabbing and touching of private body parts, and being sent unwelcome sexual pictures through digital means (Hill & Kearl, 2011). Factorial analyses (CFA and ESEM‐within‐CFA) suggest that PSH comprises two dimensions: among adolescents, visual/verbal harassment and physical harassment (Ortega et al., 2010; Vega‐Gea et al., 2016), and among early adolescents, verbal sexual aggression and general harassment (Valik et al., 2023). Some researchers exclude behaviors with physical contact in their definition of PSH (Clear et al., 2014), but most researchers, especially those studying children and adolescents, include behaviors with some physical contact (e.g., grabbing) but exclude more severe forms of sexual violence, such as rape (Espelage et al., 2015). The inclusion of both verbal and (some) physical behaviors aligns with the definition provided by the Swedish National Agency of Education (2025a). Accordingly, in this study of early Swedish adolescents, we define PSH as unwanted sexual attention from peers expressed in verbal, physical, and visual forms (e.g., receiving unwelcome sexual pictures through digital means).

### Comparing the constructs of peer victimization and peer sexual harassment

**FIGURE 1:**
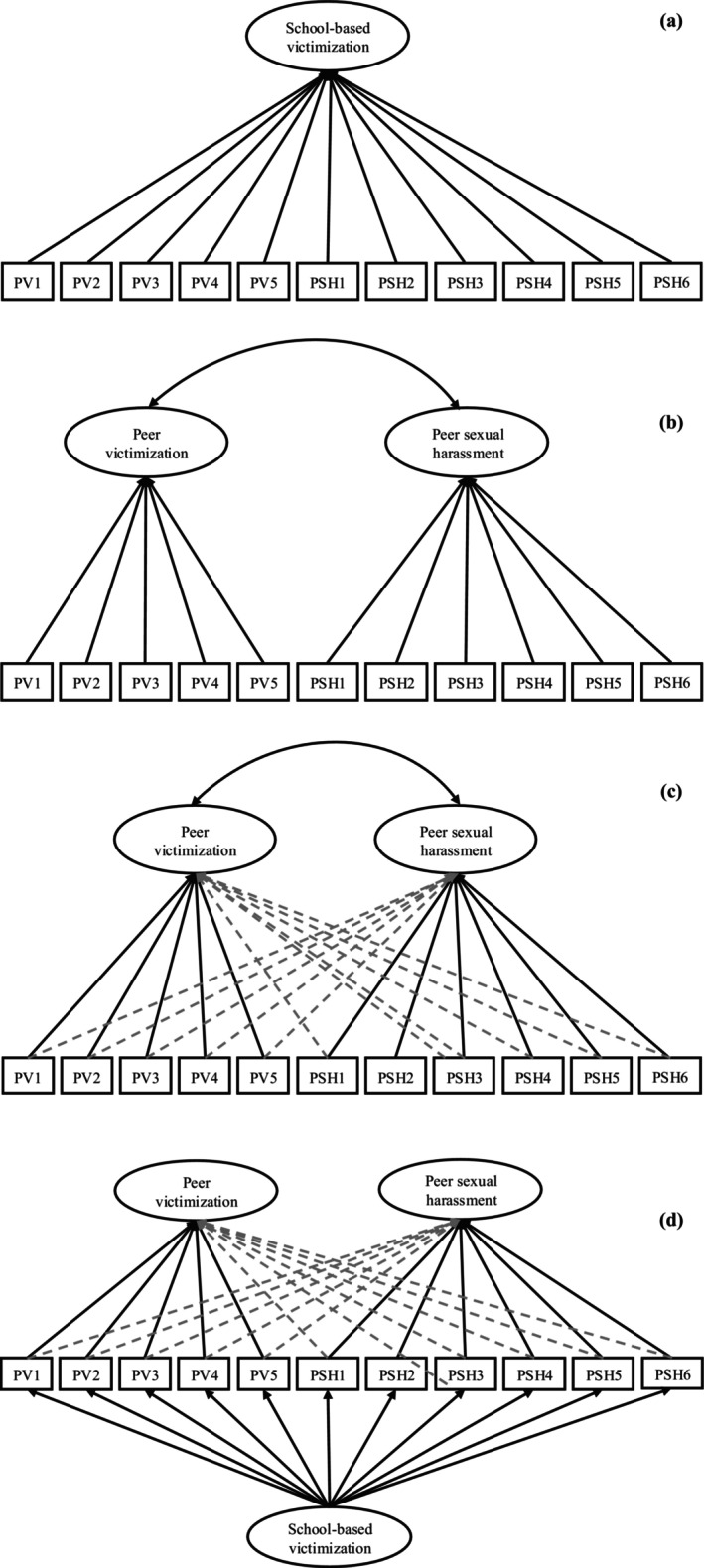
Full‐lined arrows represent target loadings, and dashed arrows represent nontarget loadings (cross‐loadings). Graphical representations for the one‐factor confirmatory factor analysis model (a), two‐factor confirmatory factor analysis model (b), two‐factor model using exploratory structural equation modeling (ESEM, c), and a bifactor ESEM model (d).

Theoretically, the constructs of peer victimization and PSH have several overlapping features (Nickerson et al., 2022). For example, both constructs involve being exposed to different forms of unwanted peer behaviors. Unwanted verbal behaviors such as homophobic and misogynistic name‐calling could be captured both by measurements assessing peer victimization, asking about teasing or hurtful name‐calling in general (e.g., Rigby, 1999), and by measurements assessing PSH, asking specifically about homophobic and misogynistic name‐calling (e.g., Hill & Kearl, 2011; Valik et al., 2023). In line with this, homophobic name‐calling is described as one of the most frequent types of verbal bullying (Espelage et al., 2018), as well as one of the most frequent types of verbal PSH (Valik et al., 2024). Research further shows that the peer victimization that students encounter is often sexualized in nature (Shute et al., 2008), and the use of the term *sexual bullying* (i.e., bullying that is sexualized, about sexuality, or about gender expression; Turner‐Moore et al., 2022) further ties the PSH construct to the peer victimization and bullying constructs. This reasoning would support that peer victimization and PSH may be viewed as one joint construct (see Figure 1a for a visual representation).

At the same time, PSH is also distinct from peer victimization and could be argued to be a construct of its own. Most importantly, PSH has a gendered and sexual dimension which is not part of the definition of peer victimization. Gruber and Fineran (2016) point out that, because of the gendered and sexual nature of PSH, it is more “directly related to hegemonic masculinity and therefore taps into potent structural and culturally sanctioned stereotypes and power relationships (masculine–feminine, heterosexual–homosexual) that are central components of social stratification” (p. 114; see also Stein & Taylor, 2023). Subsuming PSH under the rubric of bullying (as for example in the term sexual bullying) is, they argue, problematic since it increases the risk that victimization related to gender or sexuality is interpreted as private or interpersonal troubles experienced by unfortunate students, when it is actually an expression of gender power structures and illegal discrimination (Gruber & Fineran, 2008, 2016). Several researchers make a distinction between sexual and nonsexual forms of peer victimization and argue for the importance of examining them separately (Buchanan & McDougall, 2021; Gruber & Fineran, 2008; Stein & Taylor, 2023).

In line with this, it is common that researchers treat bullying or peer victimization and PSH as separate constructs (see Carnelius & Dennhag, 2023; Espelage et al., 2018; Mennicke et al., 2021; van Beusekom et al., 2020; Wang et al., 2024). The, to the best of our knowledge, only factorial examination of peer victimization and (at least one aspect of) PSH was conducted by Espelage et al. (2018). They examined whether bullying perpetration at age 10–14 years (n.b. using a bullying measure that did not encompass the bullying criteria of intention to harm, repetitive actions, and an imbalance of power) predicted homophobic name‐calling perpetration across 2 years. They were able to show through CFAs that bullying perpetration and homophobic name‐calling perpetration served as separate latent constructs. Although their study examined only the perpetrator role, only one aspect of PSH (i.e., homophobic name‐calling), and did not examine whether the findings (i.e., factorial structure) remained stable over time and had similar implications for boys and girls, their findings indicate that PSH is a separate construct from peer victimization. Altogether, the above propositions and findings support that peer victimization and PSH should be viewed as two distinct, yet to some extent related, constructs (see Figure 1b [CFA framework] and Figure 1c [ESEM framework]).

Finally, another alternative could be that peer victimization and PSH are two dimensions of an overall (i.e., “global”) victimization construct — here referred to as school‐based victimization (Marx & Kettrey, 2016) — yet they also have unique and meaningful aspects that can be separated and observed (see Figure 1d). According to Marx and Kettrey, school‐based victimization encompasses bullying, harassment (including sexual harassment), and the engendering of fear. Both peer victimization and PSH can therefore be considered distinct forms of school‐based victimization. Meantime, there may be meaningful aspects of peer victimization and PSH that are unique to each of them that can be separated and observed. We have already mentioned the gendered and sexual nature of PSH relative to peer victimization. Like peer victimization, PSH may involve intentional acts of aggression (e.g., derogation of peers deviating from dominant gender norms), but—different from peer victimization—it may also involve immature acts of showing sexual interest in peers (Bendixen & Kennair, 2017; Valik et al., 2023). Behaviors that are not clearly intentional acts of aggression, but may have alternative explanations, may be unique to PSH.

### Time and gender as moderators

To be able to draw valid conclusions about how PSH relates to peer victimization, the factorial structures of the constructs need to hold across both time and genders. Ensuring such invariance strengthens the validity of the findings and allows for meaningful comparisons between the constructs. Regarding time, our study targets a large longitudinal sample of early adolescent boys and girls followed from ages 10–12 years, representing middle school (Grades 4–6) in the Swedish school system. During early adolescence, peer victimization tends to decrease (León‐Moreno et al., 2022), while PSH, with the onset of puberty, tends to increase (Petersen & Hyde, 2009). Some suggest that peer victimization may transform into PSH as adolescents enter puberty, become more interested in pursuing romantic partners, and spend more time in mixed‐gender peer groups (Wang et al., 2024). However, research shows that PSH occurs among younger adolescents as well, but it is expressed more verbally and with fewer gender differences than among older adolescents (Valik et al., 2023).

Regarding gender, girls and boys appear to experience similar overall levels of peer victimization and PSH (Gruber & Fineran, 2008; Valik et al., 2023), but they are exposed to different types of these behaviors. For peer victimization, girls are more exposed to relational victimization, and boys are more exposed to overt victimization (Cho et al., 2022). For PSH, girls are more exposed to unwanted sexual comments, sexual pictures, sexual rumor‐spreading, and physical intimidation (Hill & Kearl, 2011). Boys are more exposed to homophobic name‐calling (Valik et al., 2023; van Beusekom et al., 2020). These age and gender differences could indicate that the constructs of peer victimization and PSH relate to one another differently across time and genders (and thereby threaten the validity of comparisons between the constructs); however, since no previous study has examined this, it needs yet to be tested.

### Aim

The emergence of the PSH construct has resulted in debate regarding its relation to the peer victimization construct. Still, we have found only one factorial examination (CFA) of peer victimization and (one aspect of) PSH (Espelage et al., 2018). Notably, the CFA approach requires that each model indicator loads only on a single latent factor (i.e., unidimensionality) and that all nontarget loadings are precisely zero (Asparouhov & Muthén, 2009). This assumption may be too strict when examining potentially related constructs such as peer victimization and PSH. In such cases, scholars have demonstrated that ESEM is a powerful analytical framework that produces more accurate parameter estimates and better model fit indices than EFA and CFA (see Morin et al., 2020; Van Zyl & Ten Klooster, 2022). Thus, this study uses ESEM as an analytical framework to examine the factorial structure of two distinct yet seemingly related constructs—peer victimization and PSH—across boys and girls during early adolescence (ages 10–12). More specifically, this study will examine how PSH relates to peer victimization by testing three different ways of conceptualizing peer victimization and PSH: (1) Peer victimization and PSH are one joint construct (Shute et al., 2008; Turner‐Moore et al., 2022; Figure 1a), (2) peer victimization and PSH are two distinct, yet related constructs (Buchanan & McDougall, 2021; Carnelius & Dennhag, 2023; Espelage et al., 2018; Gruber & Fineran, 2008; Mennicke et al., 2021; Stein & Taylor, 2023; van Beusekom et al., 2020; Wang et al., 2024; Figure 1b [CFA] and Figure 1c [ESEM]), and (3) the possibility that measures/items of peer victimization and PSH overlap to some extent, which can be observed in a global factor (i.e., school‐based victimization), but they also have unique and meaningful aspects that can be separated and observed through two distinct factors representing peer victimization and PSH (Marx & Kettrey, 2016; Figure 1d). Finally, this study aims to examine whether the factorial structure of peer victimization and PSH remains stable over time and is equivalent for boys and girls.

## METHOD

### Participants

This study used annual three‐wave data from the Swedish longitudinal PRISE (Peer Relations In School from an Ecological perspective) study (Skoog et al., 2019). At T1, 997 fourth graders participated in the study (*M* age = 10.0 years, *SD* = 0.3, range 9–11 years), including 478 boys, 511 girls, six participants who categorized themselves as “other,” and two participants who did not report gender. At T2, 966 fifth graders participated (*M* age = 11.0 years, *SD* = 0.3, range 9–13 years), including 463 boys, 489 girls, nine participants who categorized themselves as “other,” and five participants who did not report gender. At T3, 879 sixth graders participated (*M* age = 12.0 years, *SD* = 0.3, range 10–13 years), including 429 boys, 429 girls, 20 participants who categorized themselves as “other,” and one participant who did not report gender. New classmates who joined the classes during the study were invited to participate. Participants were from 63 classes across 29 middle schools located in western Sweden. At T1, most participants (93%) were Swedish‐born. Only 14% had two parents born outside of Sweden, which can be seen as representative of Sweden according to Statistics Sweden (2020).

### Measures

#### Peer victimization

Peer victimization was assessed using the Swedish version of the Victim Scale (Lunde et al., 2006; Rigby, 1999). The Victim Scale consists of five items, each referring to a different form of victimization (i.e., social exclusion, teasing, hurtful name‐calling, threats of physical violence, and overt physical violence; see Table 1). Participants reported how often they had been exposed to each form of victimization on a scale from 0 = *never* to 2 = *often*. The Swedish version of the Victim Scale has shown good internal reliability in 10‐year‐old Swedish boys and girls (*α* = .72–.76; Lunde & Frisén, 2011). In this study, the scale displayed good internal reliability (T1, *α* = .78; T2, *α* = .78; T3, *α* = .76).

**TABLE 1:** Items measuring peer victimization (Victim Scale; Lunde et al., 2006; Rigby, 1999) and peer sexual harassment (PSH‐C; Valik et al., 2023).

Peer victimization (PV)
PV1. How often have you been left out of things on purpose?
PV2. How often have you been called hurtful names?
PV3. How often have you been teased?
PV4. How often have you been threatened with harm?
PV5. How often have you been hit or kicked?
Peer sexual harassment (PSH)
Has any student done any of these things to you, even though you did not want it?
PSH1. Touched your private body parts (for example, penis, vulva, bottom, or breasts)?
PSH2. Kissed or hugged you, or tried to?
PSH3. Called you homo, fag, dyke, or the like?
PSH4. Called you dick, cunt, slut, or the like?
PSH5. Commented on or joked about private body parts (for example, penis, vulva, bottom, or breasts) or about sex?
PSH6. Shown, sent, or given you pictures or messages related to nakedness or sex?

#### Peer sexual harassment

PSH was assessed using the victimization subscale of the PSH Scale—Child (PSH‐C, Valik et al., 2023). The subscale consists of six items aimed at capturing six different PSH experiences at school, including verbal, visual, and physical forms. Participants were presented with the following prompt (English translation): “The questions are about things you do not want to happen or that do not feel right. Think about how it's been in Grade 4 (5, 6). Has any student done any of these things to you, even though you did not want it?,” followed by six items (see Table 1). Participants reported how often they had been exposed to each of the situations on a scale from 1 = *never* to 4 = *several times*. The PSH‐C has displayed good psychometrics (construct validity, internal consistency, response rate, and convergent validity) and is well suited for use among early adolescents (Valik et al., 2023). In this study, the subscale showed good internal reliability (T1, *α* = .78; T2, *α* = .69; T3, *α* = .77).

### Procedure

Schools were recruited in western Sweden during the autumn of 2019. An invitation was sent to principals of middle schools (i.e., Swedish Grades 4–6; early adolescents aged 10–12 years) in the region until the goal of *N* > 1000 early adolescents in Grade 4 participating was fulfilled. Schools of different sizes located in urban, suburban, and countryside areas were selected to represent a range of different socio‐economic backgrounds. Participants and their guardians gave active consent to participate in the study. The consent form was sent to a total of 1442 guardians, of whom 1109 gave consent, 248 did not reply, and 85 actively refused participation. Study procedures were approved by the Swedish Ethical Review Authority. The questionnaire was pilot tested with a group of early adolescents before data collection to ensure that the wording and length of the questionnaire were developmentally appropriate.

Online questionnaires were collected at school during school hours. At T1, we visited the classes to administer the survey and were available for questions. To ensure that the PSH questions were filled in correctly, a brief definition of private body parts was given, and participants were told to only report situations that had happened against their will and had occurred in the current grade. In subsequent years (due to COVID‐19), the introduction was prerecorded, and the research team was available for questions via a live chat function. Note that in Sweden, students aged 10–12 years attended school as usual during the pandemic.

Given the participants' age and the sensitive nature of the questions, we paid particular attention to ethical guidelines. Students were informed that participation was voluntary, that they could skip questions or withdraw at any time, that no unauthorized person would have access to their answers, and that results would be presented in a way that no individuals could be identified. We informed the participants that they could contact their schools' student healthcare staff if they needed someone to talk to about the issues in the survey. Participating classes received 1500 SEK (150 USD) per session for class purposes.

### Attrition and missing data analysis

Of the target sample at T1 (*N* = 973), 85% were present at T2 (*n* = 827), and 70% at T3 (*n* = 685). We performed two binomial logistic regressions (T1 vs. T2, T2 vs. T3) to examine whether the study variables (i.e., peer victimization, PSH, and gender) were systematically related to longitudinal attrition. Between T1 and T2, more PSH at T1 was significantly related to lower attrition at T2 (*p* = .044, *B* = 0.09, OR = 1.097). However, the effect size was very small, indicating that a one‐unit increase in PSH at T1 increased the odds of participating at T2 by about 1% (see Table S1). Between T2 and T3, the p‐values for the unstandardized regression coefficients ranged between .204 and .701, indicating no systematic relation between the study variables and attrition from T2 to T3 (see Table S1). Participants who did not report gender, or who categorized themselves as “other”, constituted a group too small for conducting moderation analyses and were therefore excluded prior to analysis. Further, the analytic sample used in this study comprised participants for whom we had longitudinal data across the three waves (*n* = 683, including 328 boys and 354 girls). The covariance coverage indicated that the proportion of data present for the analytical sample was very high, ranging between 98.1% and 99.1%, suggesting a low proportion of missing data.

### Plan of analysis

We used ESEM (Asparouhov & Muthén, 2009) in Mplus version 8.11. Following a sequential analytical approach (Morin et al., 2020; Van Zyl & Ten Klooster, 2022), we tested alternative competing models, including CFA, ESEM, and bifactor ESEM, to examine the factorial structure of peer victimization and PSH. This sequential process made it possible to determine the analytical approach(es) that can offer the best representation and solution in estimating peer victimization and PSH.

As part of the sequential approach, and consistent with previous arguments and findings (Shute et al., 2008; Turner‐Moore et al., 2022), we first examined a unidimensional CFA model (Model 1) in which all items measuring peer victimization and PSH were loaded onto a single latent factor (Figure 1a). Subsequently (Model 2), again using CFA and based on a priori knowledge from the literature (Buchanan & McDougall, 2021; Carnelius & Dennhag, 2023; Espelage et al., 2018; Gruber & Fineran, 2008; Mennicke et al., 2021; Stein & Taylor, 2023; van Beusekom et al., 2020; Wang et al., 2024), we modeled peer victimization and PSH as two distinct but still related latent factors. Here, items measuring peer victimization and PSH were only allowed to load on their pre‐specified respective latent factors (Figure 1b). For the third set of analyses (Model 3), we used ESEM, an analytical framework that represents a connection between traditional EFA and CFA, hence utilizing the advantages that are usually associated with both EFA and CFA (Morin et al., 2020). Using ESEM, we once again modeled peer victimization and PSH as two distinct latent factors; however, this model was less restrictive given that the items loading on their pre‐specified and distinct latent factors were also allowed to cross‐load (Figure 1c). Moreover, we used target rotation to enable items to regress on their a priori‐driven latent construct, while also allowing cross‐loading to exist, though to be as close as possible to zero (Morin et al., 2020). Finally, in Model 4 (Figure 1d), we specified a bifactor model, either in CFA or within the ESEM framework depending on the results of comparing Models 2 and 3. This model tested the presence of a global factor representing overall school‐based victimization (Marx & Kettrey, 2016), along with two distinct and specific factors representing peer victimization and PSH, respectively. For bifactor models, target (orthogonal) rotation is chosen to properly partition the variance of indicators into a global factor, reflecting shared variance among all indicators, and into specific factors reflecting unique variance among a subset of indicators not explained by the global factor.

### Model assessment and examination

The constructs used in the present study are measured using observed indicators that are ordinal and answered on a scale of four or fewer response categories. Hence, we used the weighted least squares with means and variances adjusted (WLSMV) robust estimators throughout the analyses (Flora & Curran, 2004; Rhemtulla et al., 2012). To assess model fit, we used traditional fit indices and followed the conventional cutoff points (Wang & Wang, 2019) including: (1) model Chi‐square statistics, (2) the comparative fit index (CFI >0.95), (3) the Tucker–Lewis index (TLI >0.95), (4) the root mean square error of approximation (RMSEA < 0.10), and standardized root means residuals (SRMR <0.10). Relatedly, we followed recommendations (Liu et al., 2017; Svetina et al., 2020) and findings from the relevant literature (Chen, 2007; Cheung & Rensvold, 2002) to compare competing models by inspecting a change in Δ*χ* ^2^ statistics and change in ΔCFI and ΔRMSEA as the criteria for determining a model that best represents the data. Here, a model shows a better fit if Chi‐square differences between compared models are significant (*p* < .05), and changes in CFI and RMSEA exceed ≥0.01 and ≥0.015 respectively.

In addition to assessing the model fit of the competing models, we also examined the measurement quality of the indicators to discriminate and determine the best model (Morin et al., 2020). Specifically, we evaluated the quality of indicators based on their standardized factor loadings, where factor loadings greater than .40 are generally considered an acceptable cutoff value (Wang & Wang, 2019). For model 3, where the ESEM approach was used to examine the factorial structure of peer victimization and PSH (Figure 1c) compared with a similar, though more restrictive model analyzed through traditional CFA (i.e., Model 2, Figure 1b), we also inspected the size of the correlation between the two latent factors and the size of standardized cross‐loadings. In this case, it is suggested that ESEM should be supported over CFA in case of (1) improved model fit, (2) well‐defined factors (i.e., strong loadings and loadings matching expectations), (3) reduced factor correlations, and (4) small‐to‐moderate cross‐loadings or theoretically justifiable large cross‐loadings (Morin et al., 2020). Further, it is also suggested that the presence of multiple moderate‐to‐large cross‐loadings could indicate an unmodeled global factor (e.g., higher order or bifactor model). Overall, models that meet the goodness‐of‐fit criteria and the measurement quality are deemed as well‐fitting or retained for further analyses of measurement invariance over time and across boys and girls.

### Measurement invariance

Once an appropriate model is identified, measurement invariance testing is conducted following a sequential approach involving *configural invariance, weak invariance*, *strong invariance*, *strict invariance*, *latent variances and covariances, and latent mean invariance* (Millsap, 2012) across genders (i.e., boys and girls) and time (i.e., over the three measurement occasions).

## RESULTS

Findings from the sequential analysis revealed an acceptable fit between all hypothesized measurement models (Models 1–4) and the data (see Table 2). However, a closer inspection revealed that the fit of the measurement models to the data and their respective measurement qualities differed. Specifically, results for the unidimensional model measuring peer victimization and PSH as a single unidimensional construct (Model 1) indicated an adequate fit to the data in terms of produced fit indices (CFI and TLI > .95, and RMSEA ≤ .08) and measurement quality, with all the standardized item loadings (*λ*) within an acceptable range (*λ* = 0.526–0.875). However, indications of model improvement were observed already in the two‐factor CFA model (i.e., Model 2, peer victimization and PSH are modeled as distinct, yet related latent factors) in terms of model fit (Δ*χ* ^2^ = −117.274, ΔCFI = +.028, ΔRMSEA = −.033), an acceptable range of standardized item loadings for peer victimization (*λ* = 0.549–0.828) and PSH (*λ* = 0.601–0.907), and a strong factorial relationship (*r* = .823).

**TABLE 2:** The goodness‐of‐fit statistics for the hypothesized models and for measurement invariance across gender and time.

Models type	*X* ^*2*^ (*df*)	*CFI*	*TLI*	*RMSEA* [90% CI]	Δ *X* ^*2*^ (*df*)	Δ *CFI*	Δ *TLI*	Δ *RMSEA*
Estimated model fit statistics								
Model 1 (One‐factor CFA)	212.378 (44)***	.959	.948	.075 [.065; .085]	–	–	–	–
Model 2 (Two‐factor CFA)	95.104 (43)***	.987	.984	.042 [.031; .054]	–	–	–	–
Model 3 (ESEM)	86.914 (34)***	.987	.979	.048 [.035; .060]	–	–	–	–
Model 4 (Bifactor ESEM)	30.206 (25)	.999	.997	.017 [.000; .037]	–	–	–	–
Measurement invariance fit statistics								
Gender invariance								
Configural invariance	81.321 (68)	.997	.995	.024 [.00; .042]	–	–	–	–
Weak invariance	128.381 (86)**	.990	.987	.038 [.023; .051]	42.834* (18)	−.007	−.008	+.014
Strong invariance	142.416 (101)**	.990	.989	.035 [.020; .047]	19.431 (15)	.000	+.002	−.003
Strict invariance	158.844** (112)	.989	.989	.035 [.021; .047]	19.099 (11)	−.001	.000	.000
Latent variance–covariance	134.389 (115)	.995	.996	.022 [.000; .037]	1.601 (3)	+.006	+.007	−.013
Latent means	164.745 (117)**	.989	.989	.035 [.021; .046]	12.359 (2)**	−.006	−.007	+.013
Time invariance								
Configural invariance	570.011 (435)***	.986	.984	.021 [.016; .026]	–	–	–	–
Weak invariance	623.562 (471)***	.985	.983	.022 [.017; .026]	62.027 (36)**	+.001	−.001	−.001
Strong invariance	677.779 (501)***	.982	.981	.023 [.018; .027]	67.130 (30)***	+.001	−.003	−.002
Strict invariance	704.449 (523)***	.982	.982	.023 [.018; .027]	39.968 (22)*	.000	+.001	.000
Latent variance–covariance	784.350 (529)***	.974	.974	.027 [.023; .030]	35.57 (6)***	+.008	+.008	+.004
Latent means	966.397 (533)***	.956	.957	.034 [.031; .038]	110.105 (4)***	+.018	+.019	+.007

Abbreviations: 90% CI, 90% confidence interval of RMSEA; *CFI*, comparative fit index; *df*, degrees of freedom; *RMSEA*, root mean square error of approximation; *TLI*, Tucker–Lewis index; Δ *χ* ^*2*^, Chi‐square difference test based on the Mplus DIFFTEST function for WLSMV estimation; Δ, change in fit indices from the preceding model; *χ* ^2^, model Chi‐square test statistics.

* *p* < .05;

** *p* < .01;

*** *p* < .001.

Subsequently, we examined the two‐factor model within the ESEM framework (i.e., Model 3, peer victimization and PSH are modeled as distinct, yet related latent factors, and cross‐loadings are allowed). Results revealed minor improvements/changes in model fit (Δ*χ* ^2^ = −8.190, ΔCFI = .000, ΔRMSEA = +.006), still an acceptable range of standardized item loadings for peer victimization (*λ* = 0.576–0.924) and PSH (*λ* = 0.709–0.856), and a strong, but slightly reduced relationship between the two latent factors (*r* = .795). Furthermore, the results also revealed the presence of a significant cross‐loading, more specifically, from an item expected to measure peer victimization (PV2 about hurtful name‐calling), showing a small yet considerable cross‐loading (*λ* = .25) on the PSH factor.

At this stage, findings suggested that the two‐factor model solution within CFA and ESEM frameworks fits the data better and offers better measurement quality than the unidimensional CFA model measuring victimization as a single construct. Hence, in the next step, we compared the fit indices and measurement quality of the two‐factor model within the ESEM framework (Model 3) to the corresponding model within the CFA framework (Model 2). The findings indicate that the two‐factor model within the ESEM solution offers: (1) a slightly improved model fit to the data (see Table 2); (2) a reduced latent factorial correlation between peer victimization and PSH; (3) the possibility of observing and identifying cross‐loadings (see Table 3); and (4) two distinct factors that are well‐defined. Scholars have argued that, in general, these criteria indicate that an ESEM approach offers a more correct solution than CFA (Morin et al., 2016; Van Zyl & Ten Klooster, 2022). Especially, the flexibility in allowing cross‐loadings is important to consider, as it will result in more accurate fit indices and less biased estimates such as the correlation between latent factors. This is observed in the ESEM solution (i.e., Model 3), such that the estimated latent factor correlations are reduced compared with those produced in the CFA solution (i.e., Model 2).

**TABLE 3:** Item‐level descriptive statistics, standardized factor loadings, and standard errors for models 1–4.

Items	Mean (*SD*)	Model 1: One‐factor CFA model	Model 2: Two‐factor CFA model		Model 3: Two‐factor ESEM model		Model 4: A bifactor ESEM model		
			Peer victimization	Peer sexual harassment	Peer victimization factor	Peer sexual harassment factor	Global victimization factor	Peer victimization factor	Peer sexual harassment factor
		*λ* (*SE*)	*λ* (*SE*)	*λ* (*SE*)	*λ* (*SE*)	*λ* (*SE*)	*λ* (*SE*)	*λ* (*SE*)	*λ* (*SE*)
PV1	.364 (.558)	.526 (.041)	.549 (.041), .302		.576 (.103)	*−.023 (.105)*	.433 (.051)	.354 (.065)	. *043 (.072)*
PV2	.336 (.582)	.783 (.027)	.824 (.027), .679		.579 (.068)	.*252 (.069)*	.719 (.034)	.338 (.050)	*−.036 (.051)*
PV3	.469 (.639)	.778 (.023)	.814 (.022), .663		.924 (.066)	*−.101 (.066)*	.642 (.038)	.549 (.056)	*−.086 (.049)*
PV4	.213 (.492)	.789 (.031)	.828 (.031), .685		.743 (.079)	. *092 (.083)*	.686 (.043)	.444 (.059)	. *020 (.052)*
PV5	.440 (.635)	.792 (.025)	.825 (.025), .680		.879 (.065)	*−.048 (.069)*	.648 (.039)	.549 (.051)	. *076 (.051)*
PSH1	1.207 (.577)	.731 (.037)		.761 (.037)	. *057 (.100)*	.709 (.090)	.733 (.047)	. *079 (.051)*	.355 (.087)
PSH2	1.277 (.670)	.572 (.047)		.601 (.047)	*−.126 (.122)*	.726 (.115)	.593 (.058)	*−.033 (.046)*	.451 (.107)
PSH3	1.421 (.794)	.850 (.020)		.874 (.020)	. *034 (.066)*	.850 (.061)	.884 (.027)	*−.009 (.050)*	−.167 (.095)
PSH4	1.426 (.813)	.875 (.018)		.907 (.018)	. *113 (.060)*	.801 (.057)	.895 (.025)	. *050 (.045)*	−.162 (.085)
PSH5	1.248 (644)	.721 (.039)		.751 (.040)	*−.047 (.087)*	.800 (.079)	.752 (.045)	*−.009 (.051)*	.270 (.089)
PSH6	1.053 (.317)	.716 (.067)		.747 (.068)	*−.111 (.138)*	.856 (.125)	.775 (.063)	*−.088 (.085)*	−.020 (.146)

*Note*: Items PV1‐PV5 represent a priori target loadings expected to measure peer victimization, and PSH1‐PSH6 are expected to measure peer sexual harassment. Nontarget loadings (cross‐loadings) are *italicized*. Nonsignificant loadings are underlined.

Abbreviations: *SD*, standard deviation; *SE*, standard error; *λ*, standardized factor loadings.

Thus, based on our findings and in line with recommendations from the literature (Swami et al., 2023), we decided to retain and move on with the two‐factor model tested within the ESEM framework to complete the final step of the sequential analyses. Specifically, using a bifactor ESEM solution, we investigated the presence of a global factor representing overall school‐based victimization and two distinct latent factors representing peer victimization and PSH. It is important to highlight that the examination of a global victimization factor, in addition to two distinct forms of victimization (i.e., peer victimization and PSH), is partly driven by arguments from the literature indicating that school‐based victimization encompasses both bullying and harassment (also sexual; Marx & Kettrey, 2016). However, results from the two‐factor ESEM model did not include any medium to large cross‐loadings that could indicate a global factor, which is also an important criterion to consider in addition to conceptual arguments and empirical findings. Thus, we cautiously proceeded with a bifactor ESEM model. In this model (Model 4), all items load and cross‐load on the two distinct latent factors, in addition to the global factor. The results showed major improvement in model fit (Δ*χ* ^2^ = −56.709, ΔCFI = +.012, ΔRMSEA = +.031) and an acceptable range of standardized item loadings for the global school‐based victimization (*λ* = 0.433–0.895), peer victimization (*λ* = 0.338–0.549), and PSH latent factors (*λ* = 0.270–0.451). However, for the PSH latent factor, only three of the six items had acceptable item loadings that were significant; the remaining items (3, 4, and 6) were rather explained by the global factor (see Table 3). This is to some extent expected, as the peer victimization and PSH latent factors absorb variance in the manifest variables that is not explained by the global factor. However, changes in loading patterns have also been highlighted as a potential indication of abnormal results and improper solutions (Eid et al., 2017).

### Measurement invariance

To conduct measurement invariance testing, parameter estimates, specifically unstandardized factor loadings, from the retained bifactor ESEM model were used as starting values and re‐expressed in ESEM‐within‐CFA (Marsh et al., 2014). Findings from the first level of invariance testing (i.e., *configural invariance*) across genders revealed an acceptable model fit to the data (*χ* ^2^ = 38.294, *p* = .887, CFI = 1, RMSEA = 0 [95% CI: 0.000–0.017]), and an acceptable range of standardized item loadings for the global victimization factor for boys (*λ* = 0.471–0.907) and girls (*λ* = 0.477–0.976), and the latent factor for peer victimization across boys (*λ* = 0.254–0.593) and girls (*λ* = 0.247–0.467). Nevertheless, findings also showed that the underlying factor measuring PSH might operate differently across genders, such that different items loaded significantly for boys (items 3, *λ* = 0.292, and 4, *λ* = 0.334) and girls (items 1, *λ* = 0.414, and 2, *λ* = 0.543). Relatedly, for girls only, item 3 had a large standardized loading (*λ* = 0.976) on the global factor and a negative standardized residual variance. In such cases, recommendations include constraining the residual error variance for the indicator to a small and positive value (e.g., PSH3@0.03) or a specific value based on prior research for the model to converge (Kline, 2015; Marsh et al., 2020). There are also those who argue that the model might be too complex and not appropriate for the data (Eid et al., 2017). Given the drastic changes in both loading patterns and size of loadings of the indicators across boys and girls, as well as the improper solutions (e.g., negative residual variance), we decided to proceed with invariance testing with an alternative model (Model 3).

Specifically, the two‐factor model within an ESEM solution (Model 3) offers the best fit to the data, has good measurement quality, and the results align with the conceptual definitions of peer victimization and PSH (Buchanan & McDougall, 2021; Carnelius & Dennhag, 2023; Espelage et al., 2018; Gruber & Fineran, 2008; Mennicke et al., 2021; Stein & Taylor, 2023; van Beusekom et al., 2020; Wang et al., 2024). Results from the invariance testing fully supported this model across genders and over time (see Table 2). Specifically, findings suggested that the measurement properties of the two‐factor ESEM solution had similar implications for boys and girls and across time.

## DISCUSSION

Different propositions exist in the literature as to whether the constructs of peer victimization and PSH are branches from the same tree and/or whether they are different constructs; yet there are no empirical studies that have used the appropriate analytical tools and design to clarify this issue. In this study, we sequentially tested four theoretically informed models proposing how PSH relates to peer victimization: Model 1) Peer victimization and PSH are one joint construct, Model 2) Peer victimization and PSH are two distinct, yet related constructs (tested through CFA), Model 3) Peer victimization and PSH are two distinct, yet related constructs (tested through ESEM), and Model 4) Peer victimization and PSH overlap to some extent, which can be observed in a global victimization factor, but they also have unique and meaningful aspects that can be separated and observed through two distinct factors representing peer victimization and PSH (tested through ESEM). While all four models revealed an acceptable fit, our results suggest that Model 3 is the best to describe how peer victimization and PSH should be viewed in relation to one another.

Overall, our findings suggest that peer victimization and PSH should be viewed as distinct constructs with unique features (as suggested by Model 3) rather than as one single, joint construct (as suggested by Model 1). This aligns with the idea that PSH, because of its gendered and sexual dimension tapping into structural stereotypes and power relationships, is different from peer victimization or bullying (Gruber & Fineran, 2008, 2016; Stein & Taylor, 2023). The sexual content of the harassment is hypothesized to evoke additional shame, self‐blame, and fear in its victims (Crowley & Cornell, 2020) and this may explain why some research has found PSH to be more strongly associated with adolescents' emotional problems than peer victimization or bullying (Gruber & Fineran, 2008). Norcott et al. (2021) emphasize the importance of separating sexual forms from nonsexual forms of victimization since sexual victimization seems to have important associations also with adolescent sexual development (e.g., more sexual risk‐taking). Moreover, the potentially ambivalent experience of being a victim of PSH (i.e., distressing, but sometimes flattering; Murnen & Smolak, 2000; Petersen & Hyde, 2013) further differentiates it from peer victimization. This ambivalence may be especially salient among early adolescents who are at the onset of sexual maturation and for whom being accepted, liked, and considered attractive by peers is crucial (Brown & Larson, 2009). Accordingly, being the victim of PSH has been associated not only with a range of negative outcomes but also with positive outcomes such as higher self‐esteem, higher appearance esteem, and higher self‐perceived power in the peer group (Apell et al., 2019; Holmqvist Gattario et al., 2024; Petersen & Hyde, 2009) – links that are absent for peer victimization. Finally, and importantly, our finding that peer victimization and PSH are distinct constructs aligns with the only factorial examination of peer victimization and (one aspect of) PSH which demonstrated that they serve as separate latent constructs (Espelage et al., 2018). Our study further supports the differentiation between peer victimization and PSH, while also moving beyond previous research by examining a range of PSH behaviors, the perspective of victimization (and not only perpetration), and across time and genders.

While peer victimization and PSH were found to be distinct constructs, our analyses also clearly show that they are related to one another. The finding that Model 3 (ESEM framework, allowing for cross‐loadings between constructs) provided a better fit and measurement quality than Model 2 (CFA framework, not allowing for such cross‐loadings) indicates that aspects of peer victimization and PSH are connected. The fact that the peer victimization item of hurtful name‐calling also loaded significantly on the PSH factor suggests that the connection between the constructs may be especially salient for name‐calling. This could be expected given that name‐calling is part of the operationalizations of both peer victimization and PSH (Hill & Kearl, 2011; Rigby, 1999; Valik et al., 2023). Shute et al. (2008) noted that boys' verbal victimization of girls was, in fact, almost exclusively sexual name‐calling, thereby blurring the line between the constructs of peer victimization and PSH when it comes to name‐calling. This identified connection between the constructs may be because peer victimization and PSH are both capturing the exact same situations of name‐calling, but it could also be that exposure to one type of name‐calling (e.g., nonsexual) conceptually overlaps with exposure to another type of name‐calling (e.g., sexual). Previous findings demonstrate that those who are exposed to peer victimization are more likely to be exposed to PSH (Chiodo et al., 2009; Elipe et al., 2021). Also, being exposed to one type of victimization may have emotional, behavioral, and social consequences that could increase the risk of being exposed to another type of victimization (Finkelhor et al., 2009). More research is needed to understand in which respects peer victimization and PSH are related to one another. Such research could benefit from using measurements that cover a diverse range of sexual and nonsexual name‐calling behaviors to shed further light on the overlap between the constructs. Future research may also use multidimensional measurements of peer victimization and PSH to examine overlaps between different dimensions of the constructs.

A key aspect of the current study was examining whether the factorial structure of peer victimization and PSH remained stable over time and was equivalent for boys and girls. Model 3 fulfilled both gender and time invariance, suggesting that the proposition that peer victimization and PSH are distinct, yet related, constructs held for both boys and girls and across the early adolescent years. This is an important finding as it indicates stability in how the constructs relate to one another, which in turn is valuable to the increasing longitudinal research on these constructs. It also supports the meaningfulness of making age and gender group comparisons on and across these constructs.

### Limitations

The study has a few limitations. First, our findings respond to how peer victimization and PSH are conceptually related to one another across the period of early adolescence (in this study conceptualized as the age between 10 and 12 years), but do not necessarily apply to later adolescence. The study is conducted at the age when most participants are likely prepubertal (Skoog, 2023) and PSH behaviors (especially sexual name‐calling) are starting to appear (McMaster et al., 2002; Valik et al., 2024) alongside peer victimization. PSH among postpubertal adolescents may be more sexualized (more driven by sexual intent; Forber‐Pratt & Espelage, 2018) and thereby more distinct to peer victimization. Notably, our findings showed that peer victimization and PSH were distinct constructs already at the age of early adolescence. Possibly, however, the extent to which they relate to one another (e.g., in terms of name‐calling) may change across adolescence.

A second limitation is that this study did not differentiate between PSH that occurred between same‐ versus cross‐gender peers. McMaster et al. (2002) proposed that same‐ and cross‐gender PSH are different phenomena with distinct underlying mechanisms. Schnoll et al.'s (2015) longitudinal study showed that being bullied was prospectively associated with being exposed to same‐gender but not cross‐gender PSH, potentially indicating that same‐gender PSH is more related to peer victimization as a construct. Future factorial examinations may differentiate between cross‐ and same‐gender PSH to discern whether this is true.

A third potential limitation concerns our choice of measurements. We chose measurements of peer victimization and PSH that have been used and validated in the targeted age group (Lunde & Frisén, 2011; Rigby, 1999; Valik et al., 2023). However, it is possible that the use of other measurements, assessing other situations of victimization and harassment (e.g., unwanted appearance‐related comments and rumor‐spreading), would have yielded different results in terms of which model that best represented how peer victimization and PSH relate to one another. Researchers could conduct factorial examinations similar to ours while choosing other measurements to investigate whether current findings can be replicated.

Finally, this study was conducted in a Swedish cultural context, and the findings may differ from other cultural contexts. According to Swedish laws, schools are legally obliged to take active measures against peer victimization (the Swedish Education Act [2010:800]) and PSH (the Swedish Discrimination Act [2008:567]). Also, education concerning sexuality, puberty, consent, gender equality, and healthy relationships should be integrated into the teaching of various school subjects in Swedish middle schools (the Swedish National Agency for Education, 2025b). This targeted focus could potentially influence how Swedish early adolescents respond to questions about peer victimization and PSH and thereby influence the findings of this study. Future research could examine if they can be replicated in other cultural contexts.

### Conclusion and implications

This study is the first to demonstrate that for both girls and boys and across the early adolescent years, PSH is best understood as a separate—yet related—construct from peer victimization. The relation between the constructs seems to be most salient for verbal name‐calling. The findings contribute to a better understanding of the ways in which young individuals experience harm and adversity in the context of peer relations. The findings have key implications for both research and school practices and policies. Future research in the field should carefully consider the nature of the relations between the different constructs and design studies accordingly. For example, it is likely beneficial to include measures of both peer victimization and PSH to gain a more comprehensive view of early adolescents' adverse peer experiences. Additionally, future research could use more elaborate questions regarding verbal name‐calling or explore verbal name‐calling qualitatively to further examine this identified overlap between the constructs. Concerning school practices and policies, our findings suggest that PSH should receive attention as a distinct focus in schools, as it is not necessarily being targeted in bullying or general peer victimization interventions (Gruber & Fineran, 2008). Although certain parts of existing interventions toward peer victimization (e.g., training teachers to create a supportive classroom climate and empowering bystanders to intervene; Marsh, Reeve, et al., 2023), could also reduce PSH, more targeted interventions against PSH may be needed (see e.g., Carmo et al., 2025; Taylor et al., 2017).

## AUTHOR CONTRIBUTIONS

**Kristina Holmqvist Gattario:** Conceptualization, Investigation, Funding acquisition, Writing – original draft, Writing – review and editing, Project administration, and Methodology. **Andrea Valik:** Conceptualization, Investigation, Writing – original draft, Methodology, Writing – review and editing, Formal analysis, and Data curation. **Carolina Lunde:** Conceptualization, Investigation, Funding acquisition, Writing – original draft, Methodology, Writing – review and editing, and Project administration. **Therése Skoog:** Conceptualization, Investigation, Funding acquisition, Writing – original draft, Methodology, Writing – review and editing, and Project administration. **Darun Jaf:** Conceptualization, Writing – original draft, Methodology, Visualization, Writing – review and editing, Formal analysis, and Data curation.

## FUNDING INFORMATION

This study was supported by grants from the Swedish Research Council for Health, Working Life, and Welfare (grant number 2017–00878).

## CONFLICT OF INTEREST STATEMENT

Authors have no conflicts of interest to disclose.

## ETHICS STATEMENT

Study procedures were approved by the Swedish Ethical Review Authority (April 2019, reference number 2019–02755). Participants and their guardians gave active consent to participate in the study.

## Supporting information


Table S1.

## Data Availability

The data that support the findings of this study are available on request from the corresponding author. The data are not publicly available due to privacy or ethical restrictions.
