# Supplementary material for: Peer victimization and peer sexual harassment across early adolescence: Branches from the same tree or free‐standing constructs?
Source: J Res Adolesc. 2025 Oct 3;35(4):e70079. doi: 10.1111/jora.70079 (PMC12492469; doi:10.1111/jora.70079)
Supplement: Supplementary file 1 — Table S1. [file JORA-35-0-s001.docx]

**Supplemental Table S1**

*Attrition Analysis*

| *Variables* | *B* | *SE* | *Z* | *p* | | *OR* [CI] |
| --- | --- | --- | --- | --- | --- | --- |
| T1 to T2 |  |  |  |  |  | |
| Gender T1 | 0.0841 | 0.1834 | 0.459 | 0.646 | 1.088 [0.759 – 1.56] | |
| Peer sexual harassment T1 | 0.0925 | 0.0459 | 2.015 | 0.044 | 1.097 [1.003-1.20] | |
| Peer victimization T1 | -0.0995 | 0.0512 | -1.944 | 0.052 | 0.905 [0.819-1.00] | |
| T2 to T3 |  |  |  |  |  | |
| Gender T2 | 0.0773 | 0.1547 | 0.500 | 0.617 | 1.080 [0.798 – 1.46] | |
| Peer sexual harassment T2 | -0.0135 | 0.0350 | -0.384 | 0.701 | 0.987 [0.921-1.06] | |
| Peer victimization T2 | -0.0572 | 0.0450 | -1.269 | 0.204 | 0.944 [0.865-1.03] | |
| *Note.* Estimates represent the log odds of those who remained in the study on both T1 and T2 (1 = stayed) and those who were missing at the second data collection (0 = attrition), and those who remained in the study on both T2 and T3 (1 = stayed) and those who were missing at the third data collection (0 = attrition). *T1 to T2*: *R*^2^ indices = .006 (Cox-Snell), .010 (Nagelkerke). Model χ^2^ (3) = 5.40, p = .145. Hosmer – Lemeshow χ^2^ (7) = 8.719, p = .273. *T2 to T3*: *R*^2^ indices = .005 (Cox-Snell), .007 (Nagelkerke). Model χ^2^ (3) = 4.23, p = .238. Hosmer – Lemeshow χ^2^ (8) = 5.960, p = .652. *B* = adjusted coefficient of the regression; *SE* = standard error of estimate; *OR* = Odds ratio; CI = 95% confidence interval. | | | | | | |
|  | | | | | | |
